# Enhancement of cucumber resistance under salt stress by 2, 4-epibrassinolide lactones

**DOI:** 10.3389/fpls.2022.1023178

**Published:** 2022-11-09

**Authors:** Xianxia He, Zilong Wan, Ning Jin, Li Jin, Guobin Zhang, Jian Lyu, Zeci Liu, Shilei Luo, Jihua Yu

**Affiliations:** ^1^ College of Horticulture, Gansu Agricultural University, Lanzhou, China; ^2^ State Key Laboratory of Arid land Crop Science, Gansu Agricultural University, Lanzhou, China

**Keywords:** cucumber, salt stress, 2, 4-epibrassinolide, antioxidant, MAPK, SOS

## Abstract

This study investigated the effects of exogenous 2, 4-epibrassinolide lactone (EBR) on the growth, photosynthetic pigments, antioxidant defense system, ion homeostasis, MAPK cascade and key genes of SOS signaling pathway of cucumber seedlings under salt stress using cucumber “Xinchun 4” as the test material. The experiment was set up with four treatments: foliar spraying of distilled water (CK), 50 mmol.L^-1^ NaCl (NaCl), 50 mmol.L^-1^ NaCl+foliar spray of 0.02 μmol.L^-1^ EBR (EBR+NaCl), and 50 mmol.L^-1^ NaCl+foliar spray of 24 μmol.L^-1^ Brassinazole (BRZ) (BRZ+NaCl). The results showed that EBR+NaCl treatment significantly increased plant height, above-ground fresh weight, total root length, total root surface area, average rhizome and photosynthetic pigment content compared to NaCl treatment. Meanwhile, compared with NaCl treatment, EBR+NaCl treatment significantly increased superoxide dismutase, catalase and ascorbate peroxidase (SOD, CAT and APX) activities, significantly promoted the accumulation of osmoregulatory substances (soluble sugars and proline), and thus effectively reduced malondialdehyde (MDA) content and relative electrical conductivity of cucumber leaves. Exogenous spraying of EBR also significantly reduced Na^+^/K^+^ under NaCl stress, effectively alleviating the toxic effects of Na^+^ ions. In addition, exogenous EBR induced the up-regulated expression of *CsMAPK3*, *CsMAPK4*, *CsMAPK6* and *CsMAPK9* genes in the MAPK cascade signaling pathway and *CsSOS1*, *CsSOS2* and *CsSOS3* genes in the SOS signaling pathway to enhance salt tolerance in cucumber under NaCl stress. Therefore, exogenous spraying EBR may effectively reduce the damage of salt stress on cucumber seedlings by improving antioxidant capacity, maintaining ion homeostasis and activating salt-tolerant related signaling pathways, which might promote the growth of cucumber seedlings and the establishment of root system morphology. This study provides a reference for EBR to improve the salt tolerance of cucumber.

## 1 Introduction

Salt stress is the main abiotic stress limiting plant growth and development (Luo et al., 2017). Due to excessive use of chemical fertilizers, unreasonable irrigation and crop rotation, secondary soil salinization is becoming increasingly serious (Gou et al., 2020). However, this is still challenging due to changes in climate conditions. But the study found that the use of exogenous substances is an optional method to improve the tolerance of crops to salt stress (Li et al., 2015). In order to adapt to soil salinization, plants show plasticity in appearance and morphology. For example, the most intuitive impact is the reduction of root system and the growth of over-ground parts. In addition, salt alkali stress can also lead to the imbalance of antioxidant system in plants, aggravate the degree of membrane lipid peroxidation, affect photosynthesis and respiration, and ultimately lead to abnormal plant growth and development (Acosta-Motos et al., 2017; Tanveer et al., 2018, Cai and Gao, 2020).

Salt stress has a negative impact on the cell division and expansion of plants. High salt stress limits the use of water, accompanied by cytotoxicity and imbalance of nutrient absorption, thus limiting the growth and development of roots, stems and leaves; In addition, sodium ions will also have a negative impact on nutrient absorption, usually leading to nutrient deficiency. The accumulation of Na in the cytoplasm or vacuole will disturb the K^+^/Na^+^ homeostasis, thereby reducing the K^+^/Na^+^ ratio in the cytoplasm; Excessive Na^+^ and Cl^-^ will lead to ion imbalance, and induce K^+^ deficiency due to cell membrane damage, resulting in blocked oxidation process in cells, leading to interference with photosynthetic mechanism, growth rate and biomass (Hafsi et al., 2017; Khoshbakht et al., 2018; Cao et al., 2019; Guo et al., 2020). Mild or moderate salt stress can increase the thickness of cell wall to reduce the accumulation of such potentially toxic ions; However, at high concentrations, it affects cell expansion and cell wall integrity, leading to damage to protective tissues, such as epidermis and endoderm (Silva et al., 2021; Sousa et al., 2021). Chlorophyll is an important component of photosynthesis mechanism, and plays an indispensable role in the biosynthesis of plant light absorption and metabolism (Kalaji et al., 2018). Exposure to abiotic stresses such as drought and salt will induce stomatal photosynthesis, reduce net photosynthetic rate, and accumulate reactive oxygen species (ROS) such as superoxide anion (O_2_^-^) and hydrogen peroxide (H_2_O_2_), ROS is a highly toxic substance, causing oxidative damage to the membrane, protein and nucleic acid in plant cells, which directly affects the flow of organic and inorganic solutes (Shahzad et al., 2018). Under long-term salt stress, the content of plant photosynthetic pigments (Chl a, Chl b and carotenoids) will decrease due to low photosynthetic efficiency (Shahzad et al., 2021). Photosynthetic pigments are closely related to the photosystem (ΦPSI and ΦPSII), photochemical quenching (Qp), non-photochemical quenching (NPQ) parameters and electron transfer rate (ETR) have been widely studied and reported due to the reduction of response to salt stress (More et al., 2019; Shahzad et al., 2021). Antioxidant metabolic pathway substances, such as SOD, POX, CAT, APX, play a key role in clearing ROS, reducing membrane damage, maintaining normal photosynthesis and intracellular ROS balance (Kang and Nam, 2016; Li et al., 2022). SOD disproportionate O_2_^-^ to H_2_O_2_, which is considered to be the first line of defense to eliminate ROS. It is strictly regulated by CAT, POD and APX. POD and CAT catalyze the effective removal of H_2_O_2_, and APX metabolizes H_2_O_2_ to H_2_O and O_2_ (Gupta and Huang, 2014; Tyagi et al., 2020).

Salt-Overly-Sensitive 1 (*SOS1*) is a plasma membrane Na^+^/H^+^ reverse transporter, which has been widely confirmed in model crops. The Na^+^ efflux is driven through the plasma membrane by the proton power generated by H^+^-ATPase to reduce the accumulation of Na^+^ in cells. When subjected to salt stress, SOS signal transduction pathway is activated, *SOS2* and *SOS3* protein kinase complexes phosphorylate *SOS1*, and self-inhibited *SOS1* is released, activating the transport domain, and playing the Na^+^/H^+^ transport function under the action of H^+^-ATPase (Quintero et al., 2011). In addition, ROS can also trigger mitogen activated protein kinase (MAPK) cascade reaction pathway. Activated MAPK can interact with other signal molecules and activate specific downstream targets such as transcription factors and other functional proteins, participate in signal transduction of salt or osmotic stress, antioxidant defense response and regulate ROS homeostasis in salt and oxidative stress (Hossain et al., 2015). Research showed that MAPK cascade can activate MAPK kinase protein through H_2_O_2_ accumulation, further promoting the phosphorylation cascade reaction of *AtMPK3* and *AtMPK6* (Pitzschke et al., 2009). Brassinolide (BR) treatment can induce the increase of NADPH oxidase activity, promote the accumulation of H_2_O_2_, further activate maize *MPK5*, and up regulate the activity of antioxidant defense enzymes (Zhang et al., 2010).

BR is an important natural steroid hormone, known as the sixth active and broad-spectrum plant growth hormone (Sharma, 2021). 2, 4-epibrassinolide (EBR) is a highly active BR homologue, which is widely used in various experimental studies and agricultural practices (Sun et al., 2015). The plants treated with exogenous EBR showed stronger growth, increased photosynthetic pigment content, photosynthetic efficiency and antioxidant enzyme activity (Sharma et al., 2016; Sirhindi et al., 2017). Recent studies have shown that BRs increase tolerance to various biotic and abiotic stresses in many plants, while regulating morphological, physiological and biochemical properties of a wide range of plants (Kagale et al., 2007)[7]. Exogenous EBR regulates endogenous hormones by activating EBR biosynthetic genes on the transcriptional level, thereby increasing the level of antioxidant enzyme capacity and reducing the excessive accumulation of reactive oxygen species (ROS) and MDA, thereby improving the growth of cucumber seedlings under stressful conditions (Azhar et al., 2017; Anwar et al., 2018). The study by Nie et al. showed that exogenous EBR significantly increased the activities of antioxidant enzymes and antioxidant contents in leaves and roots of cucumber under NaHCO_3_ stress, as well as AsA/DHA and GSH/GSSG ratios, significantly improved the redox balance of plants, reduced the level of ROS, decreased the degree of membrane lipid peroxidation, maintained root activity, and thus improved the alkali tolerance of cucumber seedlings (Nie et al., 2018). Some studies have shown that under salt stress, exogenous EBR inhibits the absorption of Na^+^ and Cl^−^, and reduces the levels of hydrogen peroxide (H_2_O_2_) and MDA to improve the growth physiology of maize under salt stress. Specifically, it improved the total chlorophyll content of maize, the accumulation of mineral elements and osmotic agents (free proline and glycine betaine), and the activity of antioxidant enzymes (Kaya et al., 2018). It is thus clear that, exogenous spraying EBR was beneficial to the growth, root morphogenesis and photosynthetic pigment accumulation of cucumber seedlings, which could effectively alleviate the damage to cell membrane and increase the antioxidant activity, thereby improving the photosynthetic capacity of leaves, maintaining osmotic regulation and ion homeostasis. At the same time, the salt tolerance of cucumber can be regulated by up-regulating the expression of some transcription factors (Sharma et al., 2013).

BRZ is an inhibitor of BR biosynthesis. It has been reported that exogenous application of EBR could increase the BRs level of plants, and application of BRZ can reduce the BRs level of plants. BRZ directly binds to DWF4 protein, inhibited BR biosynthesis by blocking the hydroxylation of C-22 hydroxylase, and effectively reduces BR content in plants (Asami et al., 2001). Zhu et al. treated tomatoes at green maturity with BR and BRZ respectively. The results of BRZ treatment were opposite to those of BR treatment. BRZ can reduce the content of soluble sugar, Vc and lycopene, and reduce the rate of ethylene production and the expression of key genes for synthesis (Zhu et al., 2015).

The above studies showed that the application of exogenous EBR was an effective strategy to improve salt tolerance of various plant seedlings including cucumber. However, there are few reports about EBR improving cucumber salt tolerance through MAPK and SOS pathways. Thus, in this experiment, cucumber “Xinchun 4” was used as the test material. The effects of exogenous EBR on growth, photosynthetic pigments, antioxidant defense system, ion homeostasis, MAPK cascade, and key genes of SOS signaling pathway in cucumber seedlings under salt stress were studied.

## 2 Materials and methods

### 2.1 Experimental material

The test cucumber variety was ‘Xinchun 4’, seed purchased from Shandong Xintai Kerun Seed Industry Co., Ltd. EBR was purchased from Beijing Solaibao Technology Co., Ltd. BRZ (an inhibitor of rapein lactone synthesis) was purchased from Shanghai Yuanye Biotechnology Co.

### 2.2 Experimental design

Cucumber seeds of uniform size and full seeds were selected, disinfected with 15% sodium hypochlorite for 15 minutes, then rinsed 4-5 times with distilled water, and finally soaked them in distilled water for 4 h. The seeds were evenly placed on a seedling tray covered with filter paper, and germination was carried out in the dark (28°C) in a constant temperature climate box. When most seeds germinated, adjusted the climate box to day/night (28°C/18°C), photoperiod was 12 h light/dark 12 h, photosynthetic photon flux density was 250 μmol.m^-2^s^-1^, relative humidity was 75%. When cotyledons fully flatten and true leaves grow out, they were planted in 1 L hydroponics pot. The cultivation nutrient solution was Hogarland nutrient solution with 4 plants per pot. The climate box was adjusted to day/night (25°C/15°C). The photoperiod was light 14 h/dark 10 h and the relative humidity is 75%. When the seedlings grow to two leaves and one heart, select the same size seedlings for treatment, each treatment repeated three times.

Four treatments were set in this experiment, foliar spraying of distilled water (CK), 50 mmol.L^-1^ NaCl (NaCl), 50 mmol.L^-1^ NaCl+foliar spray of 0.02 μmol.L^-1^ EBR (EBR+NaCl), and 50 mmol.L^-1^ NaCl+foliar spray of 24 μmol.L^-1^ BRZ (BRZ+NaCl). The salt stress of each treatment was foliar spraying, we sprayed EBR for 2 h and then transferred it to dark conditions for incubation. In order to strengthen the absorption effect of EBR, 0.1% Tween 80 was added into the spraying solution. When spraying, the positive and negative sides of all leaves were moistened with condensed water droplets without dropping. The treatment was conducted once every 24 h, and the related indexes were determined after 7 times consecutive treatments.

### 2.3 Determination of indicators

#### 2.3.1 Determination of growth indexes

The transparent ruler was used to measure the plant height (stem base to growth point) of seedlings, and national standard vernier caliper was used to measure stem diameter (cotyledon node position). Weigh the fresh weight of the aboveground and underground parts using a balance. The samples were dried at 105°C for 15 min and 75°C to constant weight. The dry weight of aboveground and underground parts was measured by analytical balance. The above-ground parts of the plants to be tested were cut off, the roots were cleaned, the root images were scanned using a root scanner (STD4800, Canada). Root morphological indexes were analyzed using root analysis software WinRHIZO 5.0 (Regent Instruments, Inc., Quebec City, Canada).

#### 2.3.2 Determination of photosynthetic pigment content

Chlorophyll a, b and carotenoid (car) contents were extracted according to the method of Song et al. (2016). Weighed 0.5 g of fresh cucumber leaves were extracted in 2 mL of 95% ethanol protected from light for 24 h, and the extract was analyzed for the amounts of Chl a, Chl b, and Car by reading absorbance at 665, 649, and 470 nm using a spectrophotometer UV-1780. Chlorophyll and Car contents were obtained according to the following equations (Zafari et al., 2020).

Chlorophyll a = (19.3 × A665 − 0.86 × A649) V/100 W

Chlorophyll b = (19.3 × A649 − 3.6 × A665) V/100 W

Total Chlorophyll = Chlorophyll a + Chlorophyll b

Car = (1000 A470 − 1.82 Chl a − 85.02 Chl b)

### 2.4 Determination of antioxidant enzyme activity, Membrane lipid peroxidation products and osmoregulatory substances

#### 2.4.1 Antioxidant enzyme activity

The determination of POD. Weigh about 0.1g of tissue, add 1 mL of extract, and grind into homogenate by ice bath. Centrifuge 8000 g at 4°C for 10 min, and take the supernatant. The absorbance values A1 at 30 s and A2 at 1 min 30 s were recorded at 470 nm.

POD (U/g. FW) = 7133 × ΔA/W (ΔA = A2-A1)

The determination of SOD. Weigh about 0.1 g of tissue, add 1 mL of extract, and grind it into homogenate by ice bath. Centrifuge 8000 g at 4°C for 10 min, and take the supernatant. Record the absorbance values of the initial four test tubes at 560 nm: A test, A control, A1 blank, A2 blank calculation.

ΔA determination = A determination - A control

ΔA blank = A1 blank - A2 blank

inhibition percentage = (ΔA Blank - ΔA determination)/ΔA blank × 100%

SOD (U/g. FW) = 11.11 × inhibition percentage/(1-inhibition percentage)/W

The determination of CAT. Weigh about 0.1 g of tissue, add 1 mL of extract, and grind it into homogenate by ice bath. Centrifuge 8000 g at 4°C for 10 min, and take the supernatant. Record the initial absorption value A1 at 240 nm and the absorption value A2 after 1 min.

CAT (U/g. FW) = 678 × ΔA/W, (ΔA = A1-A2).

Take about 0.1 g of tissue, add 1 mL of extract, and grind it into homogenate by ice bath. Then centrifugate it at 13000 g at 4°C for 20 min, take it out and place it on ice for testing. Record the absorbance value A1 after 10 S and A2 after 130 S at 290 nm. The calculation formula is: APX (μmol.min^-1.^g^-1^ FW) = 1.19 × ΔA/W (ΔA = A1 - A2)

### 2.5 Membrane lipid peroxidation products

Malondialdehyde (MDA) by thiobarbituric acid (TBA) method (Al-aghabary et al., 2005). Weighed 0.3 g of cucumber leaves were ground in 5 ml of trichloroacetic acid and the homogenate was centrifuged at 12000 g for 15 min at room temperature. The supernatant was mixed with an equal volume of thiobarbituric acid and heated for 10 min. After cooling, the mixture was centrifuged at 7500 g for 5 min. The absorbance values of the supernatant at 532 nm, 600 nm and 450 nm were measured using a UV-1800 spectrophotometer. MDA (mmol·g^-1^ FW) = [6.45*(A_532_-A_600_)-0.599*A_450_]* Vt/FW, where vt is the volume of the extract (ml) and FW is the fresh weight of the sample.

Relative conductivity was determined with reference to Zhao’s method (Zhao et al., 2009). Ten circular cucumber leaves with a diameter of 1 cm were cut and immersed in a small flask containing 40 mL deionized water, and shaken at 150 rpm for 2 h. The conductivity of the solution (L1) was measured with a conductivity meter (DDS-11A, China). Then, the solution was boiled for 15 min, cooled to room temperature, and the conductivity of the solution was measured as L2. Relative conductivity was calculated as (L1/L2) × 100%.

### 2.6 Qsmoregulatory substances

Soluble sugars were determined using anthrone colorimetric method (Mengxi et al., 2011). The fresh leaves (0.1 g) were placed in a 20 ml test tube and mixed by adding 5 ml of ethanol to the tube. After 30 min in a water bath at 85°C, the supernatant was collected; the procedure was repeated twice, and then distilled water was added to fix the volume to 10 ml. The soluble sugar content was determined at 625 nm using anthrone sulfate.

Soluble protein was determined using the Coomassie brilliant blue G-250 (Kučerová et al., 2019). Weighed 2 g of the sample, add 5 times the volume of phosphate buffer for grinding, centrifuge at 13000 g for 15 min at 4°C, take 0.1 mL of the supernatant, add 4.9 mL of Coomassie brilliant blue G-250 solution, mix well, stand for 2 min at 595 nm Under colorimetry, the protein content in the sample was calculated according to the absorbance.

Proline content was extracted and measured using the sulfosalicylic acid method (Ábrahám et al., 2010). Weighed 0.2 g of cucumber leaves, add 5 mL of 3% sulfosalicylic acid solution Grind, extract the supernatant 2 mL, add 2 mL glacial acetic acid, 4 mL acidic ninhydrin reagent and 2 mL 3% sulfosalicylic acid solution and shake well, heat the color development on a boiling water bath for 1 h, then add 4 mL toluene, measure the absorbance at 520 nm used UV-1800 spectrophotometer and calculate.

### 2.7 Determination of mineral element content

The content of K^+^, Ca^2+^, Mg^2+^, and Na^+^ ions was determined by the method referenced to Jin et al. (2022). Cucumber leaves to be tested were taken, dried and crushed, passed through a 0.25 mm sieve, and the dried leaf material (0.5 g) was decocted with sulfuric acid and hydrogen peroxide. The content of K^+^, Ca^2+^, Na^+^, and Mg^2+^ ions in the decoction solution was determined by a ZEEnit 700P atomic absorption spectrometer (Jena Analytica, Germany).

### 2.8 Measurement of gene expression

Total RNA was extracted using a plant RNA extraction kit (Tiangen). Reverse transcription was performed according to the instructions of the first strand synthesis kit of FastKing RT kit, complementary cDNAs were synthesized using 2 μl of RNA as template and primers were designed for fluorescent quantitative PCR (synthesized by Biotech, **Table 1**). Fluorescence quantification using SYBR Green kit (Tiangen). The volume of the reaction system was 20 μL, including 2 μL cDNA solution, 10 μL 2 * SuperReal PreMix Plus, 10 μM forward and reverse primers 0.6 μL, 0.4 μL 50 * ROX Reference DyeΔ and 6.4 μL distilled water. qRT-PCR was performed using LightCycler^®^ 480 II real-time fluorescent quantitative PCR instrument. Amplification program conditions: 95°C 15 min, 95°C 10 s, 60°C 30 s a total of 40 cycles. Three biological replicates were performed for each treatment. The relative expression levels of related genes were calculated by 2^-ΔΔCt^ method.

**Table 1:** Primer sequences for qRT-PCR.

Gene name	Forward primer (5'-3')	Reverse primer (5'-3')
*CsMAPK3*	GTCCTCCGATTATGCCTATTGG	TCTTAACCGCAACCATTTCGT
*CsMAPK4*	AATCGACGCCAAAAGGACAT	CTCTTTTTGGCGGCCTAATG
*CsMAPK6*	CCGTGCACCAGAGCTCTTACT	CAAGGGCTTCCGATCCATTA
*CsMAPK9*	CGTGCTCCCGAACTTTGTG	TTCCAGTAAGCATTTCCGCA
*CsSOS1*	CGGTAGCATGGTTGATTTTCG	GATTCGACCGGCTATGAGATG
*CsSOS2*	TGTGGAACCCCTGCTTATGTC	CGCACGACCAAATATCAGCTT
*CsSOS3*	CAAGGAAGAGTGGCGAAACC	TGGGAACGTGGTCGTGATATC
*Actin*	GCCCTCCCTCATGCCATTCT	TCGGCAGTGGTGGTGAACAT

### 2.9 Statistical analysis

Microsoft Excel 2020 was used data statistics and collation, One-way ANOVA (One-Way ANOVA, *P*<0.05) was performed using SPSS 22.0. Duncan’s method was used for multiple comparisons, and the results were expressed as “mean ± standard error” and plotted using Origin 2018.

## 3 Results

### 3.1 Effects of exogenous EBR on the growth of cucumber seedlings under salt stress

**Figure 1:**
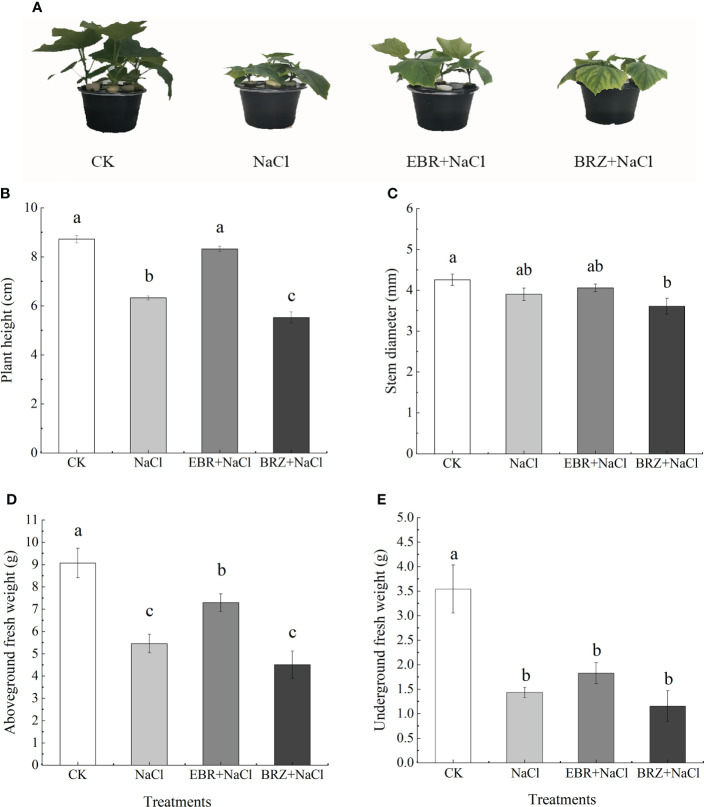
The effect of exogenous EBR on phenotype **(A)**, plant height **(B)**, stem diameter **(C)**, aboveground fresh weight **(D)** and underground fresh weight **(E)** of cucumber seedlings under salt stress. Set foliar spray with distilled water (CK) 50 mmol.L^-1^ NaCl (NaCl), 50 mmol.L^-1^ NaCl+foliar spray 0.02 μmol.L^-1^ EBR (EBR+NaCl), 50 mmol.L^-1^ NaCl + foliar spray The surface was sprayed with 24 μmol.L^-1^ BRZ (BRZ+NaCl). The treatment was conducted once every 24 h, and the related indexes were determined after 7 times consecutive treatments. Data are means ± SE of three replicates. Different letters indicate significant differences at *P* < 0.05 according to Dun’an’s multiple range test.

The growth of cucumber seedlings was inhibited under salt stress, and the plant height, stem diameter, aboveground fresh weight and underground fresh weight were significantly decreased, which were 27.1%, 8.3%, 39.8% and 59.5% lower than those of the control, respectively (**Figures 1A–E**). Compared to NaCl treatment, EBR+NaCl treatment increased 24.0%, 3.9%, 25.1%, and 21.5% in plant height, stem diameter, above-ground and below-ground fresh weight respectively. BRZ+NaCl treatment showed a decreasing trend in plant height, stem diameter, above-ground and below-ground fresh weight compared to CK and NaCl. These results indicated that exogenous application of EBR alleviated the growth inhibition of cucumber seedlings under salt stress.

### 3.2 Effect of exogenous EBR on the root system of cucumber seedlings under salt stress

**Figure 2:**
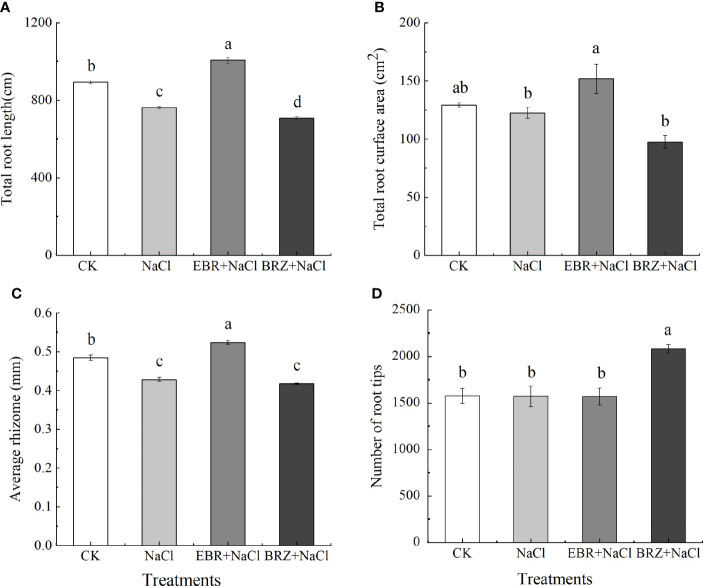
Effect of exogenous EBR on total root length **(A)**, total root surface area **(B)**, average rhizome **(C)**, and number of root tip **(D)** of cucumber seedlings under salt stress. Data are means ± SE of three replicates. Different letters indicate significant differences at *P* < 0.05 according to Duncan’s multiple range test.

**Figure 2** showed that the root growth of cucumber seedlings was inhibited under salt stress. The total root length, total root surface area, mean rootstock and number of root tips of cucumber decreased by 14.67%, 5.17%, 11.6% and 0.32%, respectively, under salt stress compared with CK (**Figures 2A–D**). Total root length, total root surface area and mean rootstock increased by 11.23%, 14.96% and 7.58%, respectively, in the EBR+NaCl treatment compared to the salt stress treatment; there was no difference in the number of root tips. Compared with NaCl treatment, the total root length, total root surface area and average rhizome of BRZ+NaCl treatment decreased by 7.2%, 20.43%, and 2.5%, respectively, and the number of root tips increased by 32.74%. These results indicated that exogenous application of EBR alleviated the growth inhibition of cucumber seedlings under salt stress.

### 3.3 Effect of exogenous EBR on the pbotosynthetic pigment content in leaves of cucumber seedlings under salt stress

**Figure 3:**
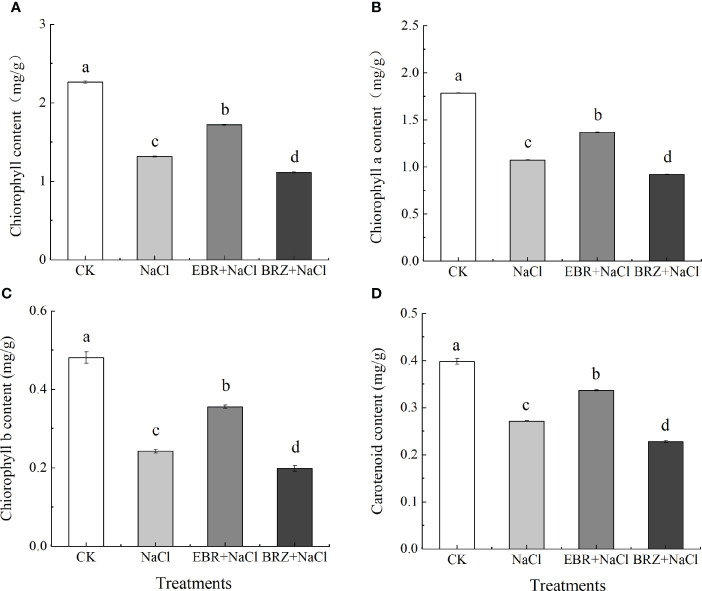
Effect of exogenous EBR on total chlorophyll content **(A)**, chlorophyll a **(B)**, chlorophyll b **(C)**, and carotenoid **(D)** content in leaves of cucumber seedlings under salt stress. Each value is the mean of three independent experiments, Data indicate the mean ± SE (*n* = 3). Significances were tested within the same day by one-way ANOVA. Different letters indicate significant differences (*P* < 0.05).

As shown in **Figure 3**, under NaCl treatment, the total chlorophyll content, chlorophyll a, chlorophyll b and carotenoid content in cucumber leaves were significantly reduced by 41.9%, 39.8%, 49.6% and 31.8% compared with CK, respectively (**Figures 3A–D**). This indicated that NaCl treatment inhibited the synthesis of photosynthetic pigments in cucumber seedling leaves. Compared with NaCl treatment, the total chlorophyll content, chlorophyll a, chlorophyll b and carotenoid content of EBR+NaCl treatment increased by 30.9%, 27.2%, 31.8%, and 24.1%, respectively. Compared with the salt treatment, the total chlorophyll content, chlorophyll a, chlorophyll b and carotenoid content of BRZ+NaCl treatment decreased by 15.2%, 14.54%, 18.0%, and 16.1% respectively, which aggravated the salt toxicity.

### 3.4 Effect of exogenous EBR on the antioxidant enzyme activity of cucumber seedling leaves under salt stress

**Figure 4:**
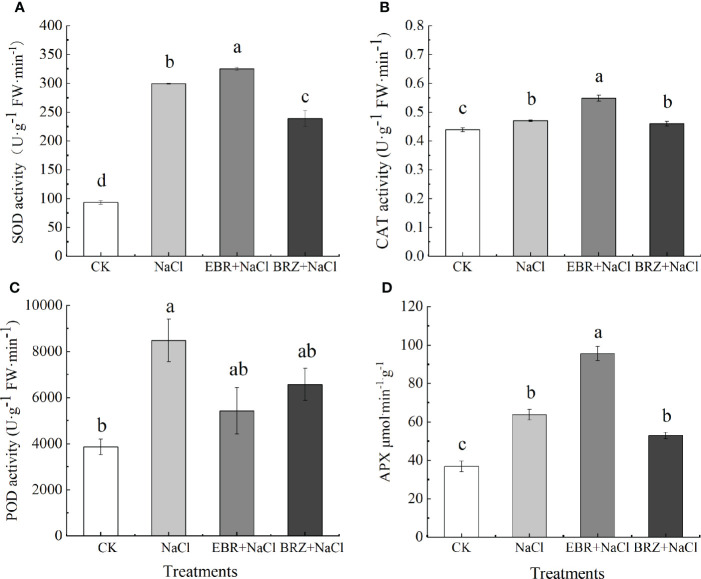
Effect of exogenous EBR on superoxide dismutase **(A)**, catalase **(B)**, peroxidase **(C)** and ascorbate peroxidase **(D)** activities in cucumber seedling leaves under salt stress. Data are means ± SE of three replicates. Different letters indicate significant differences at *P* < 0.05 according to Duncan’s multiple range test.

According to the results of antioxidant activity, the activities of SOD, CAT, POD and APX in cucumber seedling leaves were significantly increased under salt stress (**Figures 4A–D**). Compared with salt treatment, SOD, CAT and APX activities of EBR+NaCl treatment increased by 8.6%, 16.81% and 49.78%, respectively, while POD activity decreased by 35.98%. Compared with NaCl treatment, SOD and POD activities of BRZ+NaCl treatment were significantly decreased by 26.5% and 30.7%, respectively, while CAT and APX activities were not significantly decreased. This indicated that exogenous application of EBR could improve the antioxidant enzyme activity of cucumber seedling leaves under salt stress, and spraying BRZ inhibited the increase of EBR against oxidase.

### 3.5 Effects of exogenous EBR on the contents of soluble sugar, soluble protein and proline in leaves of cucumber seedlings under salt stress

**Figure 5:**
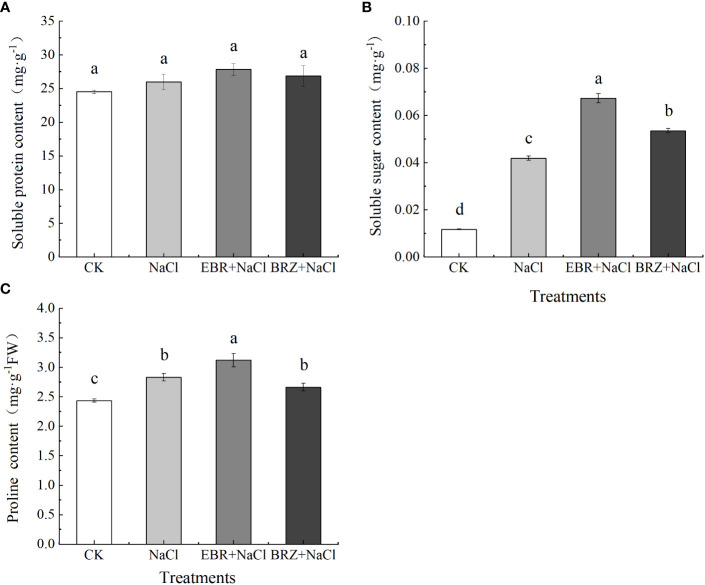
Effects of exogenous EBR on the contents of soluble sugar **(A)**, soluble protein **(B)** and proline **(C)** in leaves of cucumber seedlings under salt stress. Each value is the mean of three independent experiments, Data indicate the mean ± SE (*n* = 3). Significances were tested within the same day by one-way ANOVA. Different letters indicate significant differences (*P* < 0.05).

Compared with CK, the contents of soluble protein, soluble sugar and proline in NaCl treatment were increased by 5.7%, 75%, and 13.8%, respectively (**Figures 5A–C**). The soluble protein, soluble sugar and proline contents were increased by 7.19%, 60.6%, and 10.1%, respectively, in the EBR+NaCl treatment compared to the NaCl treatment. Compared with salt treatment, soluble protein and soluble sugar contents increased by 3.2% and 22.2%, respectively, while proline content decreased but was not significant in BRZ+NaCl treatment.

### 3.6 Effects of exogenous EBR on malondialdehyde and relatiye conductivity in leaves of cucumber seedlings under salt stress

**Figure 6:**
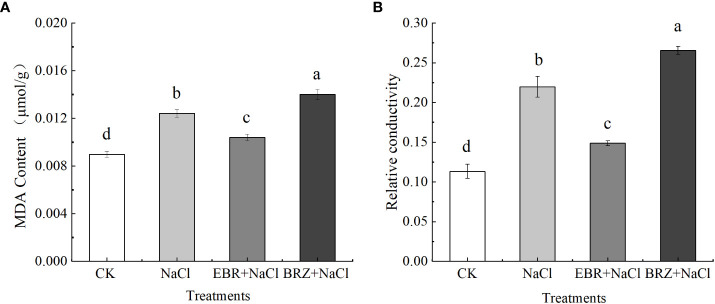
Effects of exogenous EBR on malondialdehyde **(A)** and relatiye conductivity **(B)** in leaves of cucumber seedlings under salt stress. Data are means ± SE of three replicates. Different letters indicate significant differences at *P* < 0.05 according to Duncan’s multiple range test.

The relative conductivity index and MDA content can reflect the damage degree of salt stress on plants. Our results showed that compared with the control, MDA content and relative conductivity significantly increased by 27.4% and 50% under NaCl stress (**Figures 6A, B**). Compared with NaCl treatment, the MDA content and relative conductivity of EBR+NaCl treatment decreased by 16.1% and 32.3%, respectively. After BRZ+NaCl treatment, MDA content and relative conductivity increased by 35.7% and 57.4%, respectively. This indicated that exogenous EBR could reduce the MDA content of cucumber seedling leaves, alleviate the damage of cell membrane under salt stress, and improve the salt tolerance of cucumber seedlings.

### 3.7 Effect of exogenous EBR on the ion content in leaves of cucumber seedlings under salt stress

**Figure 7:**
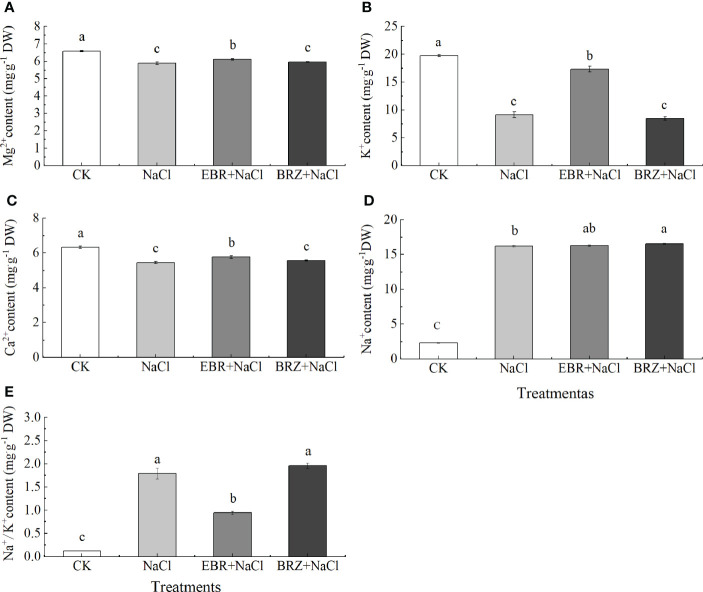
Effect of exogenous EBR on the content of Mg^2+^ **(A)**, K^+^ **(B)**, Ca^2+^ **(C)**, Na^+^ **(D)**, Na^+^/K^+^ **(E)** ions in the leaves of cucumber seedlings under salt stress. Data are means ± SE of three replicates. Different letters indicate significant differences at *P* < 0.05 according to Dun’an’s multiple range test.

The ion content in plants is regulated by various processes (such as ion absorption, transportation and accumulation), which can be used to evaluate the physiological status of plants. The measurement results were shown in **Figure 7**. Compared with CK, under NaCl treatment, the contents of Mg^2+^, K^+^ and Ca^2+^ decreased by 10.5%, 53.8%, and 13.9%, respectively, and the content of Na^+^ increased by 85.9% while the Na^+^/K^+^ ratio increased significantly. Compared with NaCl treatment, the contents of Mg^2+^, K^+^, Ca^2+^ and Na^+^ in EBR+NaCl treatment increased by 3.7%, 47.4%, 5.7% and 0.6%. However, the Na^+^/K^+^ ratio decreased significantly by 47.3%. After BRZ+NaCl treatment, Na^+^, Ca2^+^, K^+^, Mg^2+^ content and Na^+^/K^+^ ratio did not change significantly.

### 3.8 Effect of exogenous EBR on gene expression of MAPK salt resistance pathway in cucumber seedlings under salt stress

**Figure 8:**
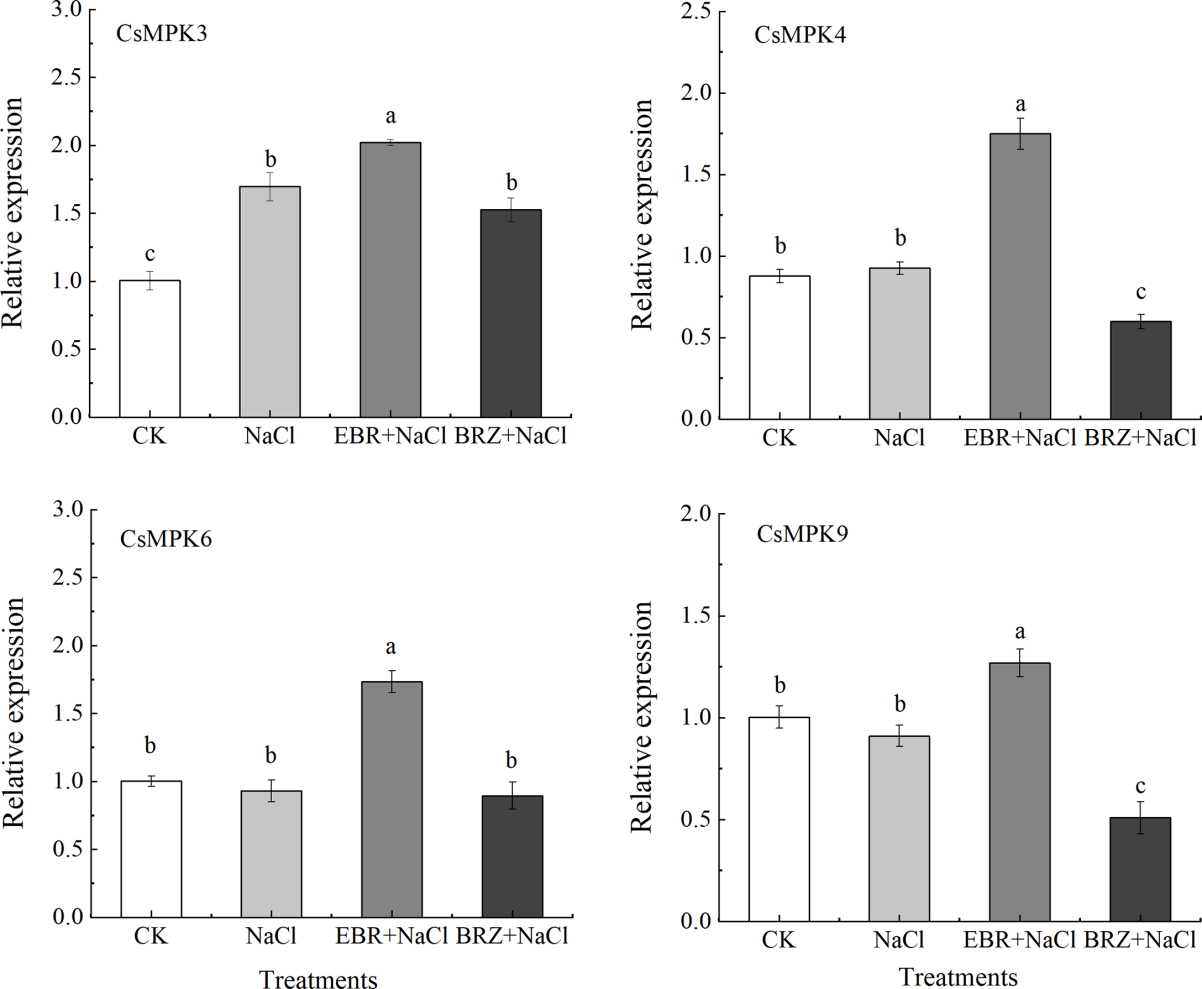
Effect of exogenous EBR on the relative expression of *CsMAPK3*, *CsMAPK4*, *CsMAPK6*, and *CsMAPK9* genes in the MAPK cascade pathway of cucumber seedlings under salt stress. With *CsActin* as the internal parameter, the relative expression was calculated by 2^-ΔΔCt^ method, and value represents mean ± SE of the three biological replications. different letters indicate significant differences at *P* < 0.05 according to Duncan’s multiple range test.

MAPK kinase cascade plays an important role in signal transduction of plant resistance to abiotic stress. As shown in **Figure 8**, the expression of *CsMAPK3*, *CsMAPK4*, *CsMAPK6* and *CsMAPK9* genes in cucumber was induced by salt stress. Compared with NaCl treatment, the expression of *CsMAPK3*, *CsMAPK4*, *CsMAPK6* and *CsMAPK9* genes in EBR+NaCl treatment were significantly up-regulated by 15.8%, 46.9%, 47.1%, and 28.3%, respectively. Under BRZ+NaCl treatment, BRZ inhibited the induction of these genes by exogenous EBR under salt stress. It could be seen that exogenous EBR can induce the expression of *CsMAPK3*, *CsMAPK4*, *CsMAPK6* and *CsMAPK9* genes at the transcription level in the MAPK cascade pathway of cucumber seedlings under salt stress, and participate in the regulation of salt tolerance of cucumber seedlings enhanced by EBR.

### 3.9 Effect of exogenous EBR on the expression of *CsSOS1*, *CsSOS2*, *CsSOS3* genes in cucumber seedlings under salt stress

**Figure 9:**
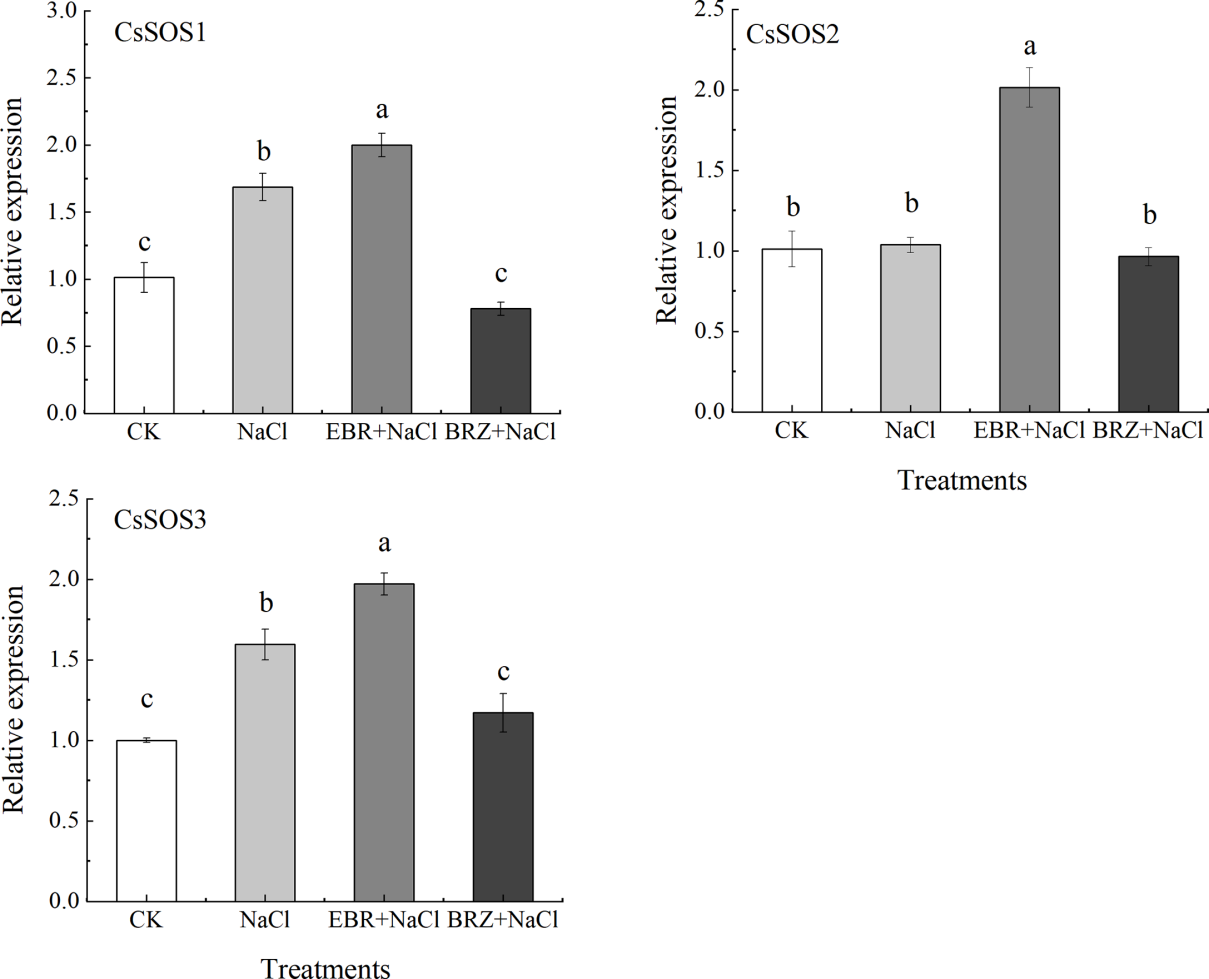
Effect of exogenous EBR on the expression of *CsSOS1*, *CsSOS2*, and *CsSOS3* genes in cucumber seedlings under salt stress. With *CsActin* as the internal parameter, the relative expression was calculated by 2^-ΔΔCt^ method, and value represents mean ± SE of the three biological replications. Different letters indicate significant differences at *P* < 0.05 according to Duncan’s multiple range test.

The SOS pathway is an important signal transduction pathway for plants to respond to salt stress signals. Compared with CK, NaCl treatment alone significantly up-regulated the expressions of *CsSOS1* and *CsSOS3* by 40.2% and 37.1%, respectively, while the *CsSOS2* gene was slightly up-regulated but not significantly (**Figure 9**). Under EBR+NaCl treatment, the expressions of *CsSOS1*, *CsSOS2*, and *CsSOS3* was significantly up-regulated by 15.7%, 48.5%, and 19.4%. BRZ+NaCl treatment could significantly inhibit the expression of *CsSOS1* and *CsSOS3*, but the difference of *CsSOS2* expression was not significant. These results suggest that exogenous spraying of EBR could protect against salt stress by regulating SOS pathway gene expression.

## 4 Discussion

The plant growth indicators are the most visual performance of the effect of stress on plant growth. The root system is an absorptive and synthetic organ that can directly perceive abiotic stresses and can largely influence seedling growth and respond to seedling condition. Many researchers have also reported deleterious effects of salt stress on plant growth and biomass (Ahmad et al., 2016; Ling et al., 2020). The reason for this may be that salt stress inhibits cell division and elongation, cells are poisoned by salt stress, cell integrity is damaged, mineral absorption is reduced, and nutrition is maladjusted (Ashraf et al., 2010; Acosta-Motos et al., 2015; Ling et al., 2020). It has been reported in the current research that salt stress leads to the decline of plant related growth indicators, such as wheat (El-Hendawy et al., 2017), sweet sorghum (Yang et al., 2020) and rice (Chang et al., 2019). Jia et al. studies have shown that BR, as an intermediate, could regulate the cell division or elongation of plant root, the initiation, emergence or elongation of lateral root (LR), and the formation of root hair (RH) (Jia et al., 2021). It was reported that EBR is considered to activate H^+^-ATPase, and H^+^-ATPase participates in the activation of cell wall loosening enzyme, regulates cell division and cell elongation, which is one of the main reasons for promoting plant growth (Ahmad et al., 2018b; Alam et al., 2019). In this study, we found that the use of EBR significantly increased the plant height, stem diameter, aboveground fresh weight, underground fresh weight and root morphological indicators of cucumber seedlings under salt stress (**Figures 1**, **2**). However, we found that BRZ had the opposite effect (**Figures 1**, **2**). Therefore, our results revealed that exogenous EBR had a potential role in alleviating the growth of cucumber seedlings under salt stress. Similar results have been reported in pepper (Abbas et al., 2013), pea (Shahid et al., 2011), tomato (Ahmad et al., 2018a), wheat (Dong et al., 2017) and other crops, and the use of exogenous EBR has also been proved to alleviate the growth and biomass of crops under salt stress.

Salt stress would lead to the inhibition of pigment degradation and pigment synthesis, which will adversely affect the chlorophyll content (Neelam and Subramanyam, 2013).The Chl a, Chl b, total chlorophyll and Car are the main light absorption, which play an important role in improving the light capture efficiency and net photosynthesis rate of plants (Wang et al., 2015). The reduction of chlorophyll content in leaves under salt stress is due to the destruction of chlorophyll pigment, the reduction of pigment synthesis and the instability of pigment protein complex (Rasool et al., 2013). Under salt stress, exogenous EBR can enhance the expression of EBR synthesis pathway, increase the activity of Calvin cycle enzyme, and improve photosynthesis (Li et al., 2016). In our study, the contents of Chl a, Chl b, total chlorophyll and Car under exogenous EBR treatment were significantly higher than those under salt stress alone (**Figure 3**). However, we found that the chlorophyll content and Car content decreased significantly after BRZ application, which indicated that exogenous EBR might promote pigment synthesis (**Figure 3**). This is similar to the previous results, exogenous EBR can increase chlorophyll and Car content (Alam et al., 2020). This may be because under salt stress, the activity of phytoene synthase (PSY) in the carotenoid biosynthesis pathway is enhanced and carotenoid biosynthesis is promoted under the condition that plants overcome oxidative stress and exogenous EBR supplementation (Sharma et al., 2016). Our research results show that EBR can improve the inhibition of photosynthesis of cucumber induced by salt stress.

Plant tolerance and adaptation to salt stress includes a complex network of physiological and biochemical responses, metabolic pathways and signaling pathways, such as enzymes and non-enzymatic antioxidants, which protect cells from oxidative damage by increasing enzyme activity to remove reactive oxygen species (Soylemez et al., 2017).In our study, salt stress increased the activities of SOD, CAT, APX and POD enzymes in cucumber seedlings (**Figure 4**); In addition, MDA and relative conductivity also increased significantly (**Figure 5**). Exogenous EBR further increased SOD, CAT and APX activities, while POD activity, MDA and relative conductivity were alleviated compared with salt stress. However, compared with EBR treatment, the activities of antioxidant enzymes and membrane lipid peroxidation products under BRZ treatment had opposite effects. This is similar to the research in tomato (Ahmad et al., 2018a), corn (AbdElgawad et al., 2016), wheat (Dong et al., 2017), etc. In the current research, SOD is considered to convert O_2_^-^ to H_2_O_2_ and O_2_, and the peroxidation process of CAT and APX converting H_2_O_2_ to H_2_O is consistent (Dong et al., 2017).This shows that exogenous EBR could protect cucumber cell membrane from oxidative damage induced by salt stress, increase the activity of antioxidant enzymes, facilitate the removal of reactive oxygen species and improve the tolerance of cucumber seedlings. Therefore, EBR can be directly related to membrane protection under salt stress.

Proline, soluble sugar and soluble protein are important penetration regulators. Proline is an effective antioxidant to eliminate ROS, which can neutralize free radicals and reduce oxidative stress, it is accumulation plays an important role in response to stress and regulation of growth and development; Soluble sugar has dual effects on ROS, which is related to both ROS metabolic pathway and NADPH production pathway, and is helpful for ROS elimination (Yusuf et al., 2017). Our study shows that exogenous EBR can significantly increase the content of proline and soluble sugar, and the difference in soluble protein is not significant (**Figure 6**). Studies on apples and peanuts also showed that exogenous EBR promoted the accumulation of proline and soluble sugar, thus maintaining osmotic balance (Su et al., 2020; Li et al., 2022). Therefore, we speculate that EBR promoted the accumulation of proline and soluble sugar, which may be related to the increase in the activity of enzymes related to proline metabolism and the expression of genes related to the synthesis pathway, to protect seedlings from osmotic stress.

Nutrient disturbance under salt stress reduces plant growth by affecting nutrient availability, transport and distribution. Research showed that under salt stress, Na^+^ and Cl^−^ compete with nutrients such as K^+^ and Ca^2+^, due to specific ionic toxicity (such as Na^+^ and Cl^−^) and ion imbalance of metabolic components, plant growth inhibition is caused (Ahmad et al., 2018a). Under salt stress, K^+^ and Ca²⁺ decreased significantly and Na^+^ content increased significantly, along with a significant increase in Na^+^/K^+^ values. EBR increased K^+^ and Ca^2+^ content led to a decrease in Na^+^/K^+^ ratio (**Figure 7**), Research showed that CDPK and Ca^2+^-ATPase are important Ca^2+^ sensors, Ca^2+^-ATPase can promote the tolerance of tobacco to salinity and drought stress by clearing ROS and enhancing the expression of stress response genes (Perochon et al., 2011). *Arabidopsis* polyploid plants enhance salt tolerance by accumulating more K^+^, less Na^+^ and higher K^+^/Na^+^ *in vivo* (Chao et al., 2013). Therefore, the inflow of high Na^+^ ions may lead to the outflow of K^+^, the imbalance of ion steady state, and the increase of ROS accumulation. The exogenous EBR increases K^+^ and Ca^2+^ content, reduce the ratio of K^+^/Na^+^, maintain the steady state of nutrient ions and regulate the synthesis and elimination of ROS to endow seedlings with salt tolerance.

MAPK signal cascade and SOS pathway are very conservative and important signal transduction modes in eukaryotes, which are involved in many processes such as regulating plant growth, development, and biological/abiotic stress (Meng and Zhang, 2013). Research showed that MAPK signal cascade pathway was involved in the signal transduction of SOS pathway, which could regulate SOS and induce SOS pathway (Pitzschke and Hirt, 2006).Yu et al. also found that PA (phosphatidic acid) could combine with *AtMPK6* to induce its activation. The activated *AtMPK6* could activate the targeted phosphorylation of *SOS1*, help to reduce the accumulation of Na^+^ in *Arabidopsis* leaves under salt stress and enhance the salt tolerance of *Arabidopsis* (Yu et al., 2010). Recent research showed that when Ca^2+^ concentration changes, *SOS3* first transmits the signal to downstream signaling proteins; *SOS3* gene activates *SOS2* through signal transduction, which is the intermediate hub of the signaling pathway, and *SOS3* forms a complex with *SOS2* and interacts with *SOS1*, and exports accumulated Na^+^ out of the cell to maintain ion homeostasis. In addition, phosphorylated *SOS2* can activate the Na^+^/H^+^ antiporter NHX1 in the membrane, which can also export the accumulated Na^+^ out of the cell and maintain ionic homeostasis (Fang et al., 2021). This study showed that, compared with salt stress, the expression of *CsMAPK3/4/6/9* and *CsSOS1/2/3* genes induced by exogenous EBR is significantly up-regulated, the content of Ca^2+^ is increased, and the growth of Na^+^ is slowed down (**Figures 7**–**9**). This shows that exogenous EBR plays a cross role in salt stress by involving MAPK cascade pathway and SOS pathway, and Ca^2+^ and Na^+^ signal transduction plays a cross role in salt stress, forming a cascade signal response, jointly regulating the expression of key genes and ion homeostasis, and regulating the salt tolerance of cucumber. Shu et al. (2022) reported that plants overexpressing tomato *MAPK3* enhanced salt tolerance, in which the expression of SOS pathway genes *SlSOS1*, *SlSOS2*, *SlSOS3* and ethylene signal pathway genes *SlACS2*, *SlEIN2* and *SlERF2* increased significantly, revealing that *SlMAPK3* responded to salt stress by clearing ROS accumulation and up regulating ethylene pathway related genes. In addition, the researchers found that exogenous melatonin and unicorn lactone could induce further up regulation of MAPK3/4/6/9 genes in cucumber under salt stress (Sun et al., 2021; Zhang et al., 2022). Therefore, the interaction mechanism of MAPK signal cascade and SOS pathway under the action of exogenous EBR to regulate the expression of related genes to enhance salt tolerance of cucumber still needs a lot of follow-up experiments to verify.

## 5 Conclusions

**Figure 10:**
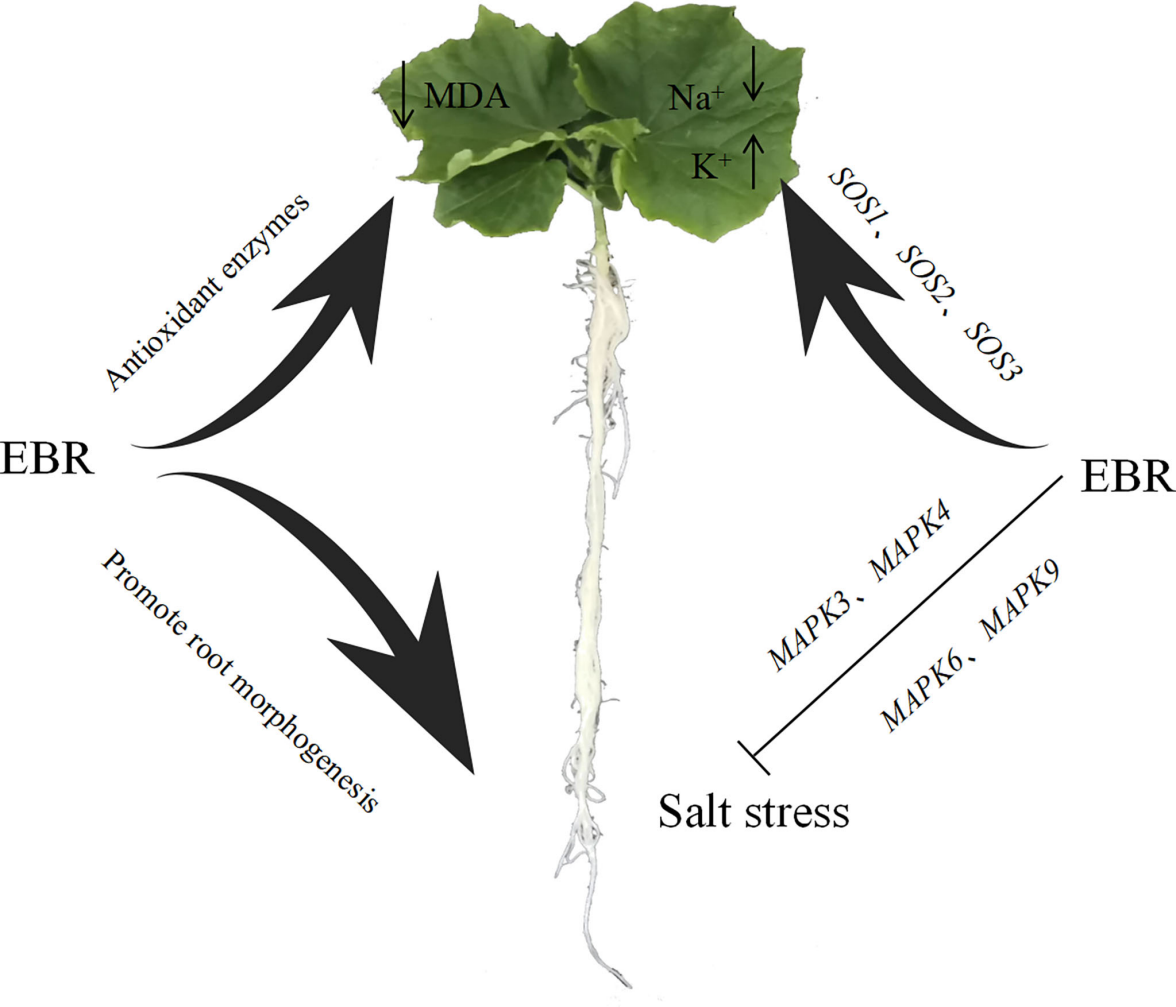
Schematic diagram for the effect of EBR on salt stress. The Figure illustrates the pathway by which EBR alleviates salt stress through different pathway.

In summary, EBR could improve the activities of antioxidant enzymes (SOD, POD, CAT and APX), promote the accumulation of osmoregulation substances (soluble protein, soluble sugar and proline), reduce the MDA content and relative conductivity of cucumber leaves, and effectively alleviate the oxidative damage of cucumber seedlings under salt stress. In addition, exogenous EBR significantly reduced Na^+^/K^+^ under NaCl stress, effectively alleviating the toxic effects of Na^+^ ions. Meanwhile, exogenous EBR induced the up-regulated expression of *CsMAPK3*, *CsMAPK4*, *CsMAPK6*, and *CsMAPK9* in the MAPK cascade signaling pathway and *SOS1*, *SOS2* and *SOS3* key genes in the SOS signaling pathway to enhance salt tolerance in cucumber under NaCl stress and a potential schematic diagram is shown in **Figure 10**. Future research will focus on how EBR regulates cucumber salt tolerance through BR signaling pathway.

## Data availability statement

The original contributions presented in the study are included in the article/supplementary material. Further inquiries can be directed to the corresponding authors.

## Author contributions

XH, ZW, SL and NJ designed the study. XH, LJ, ZW, and NJ performed the work. XH was involved with writing the manuscript. XH, NJ and ZW analyzed the data. GZ, SL, ZL, XH, JL and JY revised the manuscript. All authors contributed to the article and approved the submitted version.

## Funding

This research was supported by the Education Science and Technology Innovation Project of Gansu Province (GSSYLXM-02); Study on the Mechanism of Hydrogen Sulfide and Nitric Oxide Enhancing Salt Tolerance of Cucumber Seedlings (32160705); The Central Guide for Local Science and Technology Development Special Project (ZCYD-2021-6); Gansu Top Leading Talent Plan (GSBJLJ-2021-14); Gansu Agricultural University Scientific Research startup fund (GAU-KYQD-2020-22); Nature fund of Gansu Province (21JR7RA816).

## Conflict of interest

The authors declare that the research was conducted in the absence of any commercial or financial relationships that could be construed as a potential conflict of interest.

## Publisher’s note

All claims expressed in this article are solely those of the authors and do not necessarily represent those of their affiliated organizations, or those of the publisher, the editors and the reviewers. Any product that may be evaluated in this article, or claim that may be made by its manufacturer, is not guaranteed or endorsed by the publisher.
