# Supplementary material for: Enhancement of cucumber resistance under salt stress by 2, 4-epibrassinolide lactones
Source: Front Plant Sci. 2022 Nov 9;13:1023178. doi: 10.3389/fpls.2022.1023178 (PMC9682097; doi:10.3389/fpls.2022.1023178)
Supplement: Supplementary file 1 [file Table_1.docx]

| Table_S1 Standard curve of organic solute | |
| --- | --- |
| Name | Standard curve |
| Soluble sugar | y = 0.6019x + 0.0766 |
| Soluble protein | y = 0.00462x - 0.01 |
| Proline | y = 0.064x - 0.0019 |
